# Human Cortico‐Cerebellar Dynamics During Motor Error Processing After Stroke

**DOI:** 10.1002/hbm.70227

**Published:** 2025-05-15

**Authors:** Nitesh Singh Malan, Raghavan Gopalakrishnan, David Cunningham, Olivia Hogue, Kenneth B. Baker, Andre G. Machado

**Affiliations:** ^1^ Center for Neurological Restoration Cleveland Clinic Cleveland Ohio USA; ^2^ Center for Rehabilitation Research, MetroHealth Systems Cleveland Ohio USA; ^3^ Department of Quantitative Health Sciences Cleveland Clinic Cleveland Ohio USA; ^4^ Department of Neurosciences Lerner Research Institute Cleveland Ohio USA; ^5^ Department of Neurosurgery Cleveland Clinic Cleveland Ohio USA

**Keywords:** cortico‐cerebellar coherence, deep brain stimulation (DBS), dentate nucleus, event‐related desynchronization/synchronization (ERD/S), event‐related potentials (ERPs), granger causality and motor error processing

## Abstract

The cerebellum acts as a forward internal model to predict motor outcomes, compare them with sensory feedback, and generate prediction errors that refine prediction accuracy. Our physiological understanding of cerebellar function during motor control derives predominantly from animal experiments and clinical observations in patients with disorders of the cerebellum or its connections with the cerebrum and spinal cord. Here, we report a human electrophysiology‐based investigation of cerebello‐thalamo‐cortical pathway activity during motor error detection and correction. Participants performed a computerized motor oddball task while synchronized electrophysiological recordings were collected from cerebellar dentate (DN) using depth electrodes and scalp electroencephalography (EEG). The task involved moving a 2‐D ball on a screen toward a predetermined target at 40% (standard trials) or 20% (oddball trials) of their maximum voluntary contraction. Six participants completed an average of 239 trials, with oddball trials randomly occurring with a 30% frequency. At the cortex, oddball trials exhibited significantly greater centro‐parietal error positivity and fronto‐centro‐parietal desynchronization during error correction, predominantly in the alpha and low beta frequency bands. DN examination also revealed greater alpha and low beta desynchronization during error correction. Lastly, oddball trials showed significantly greater cortico‐cerebellar coherence during error correction in the same frequency bands with bidirectional interaction between the cortex and DN. These findings expand on the cortico‐cerebello‐cortical physiology of human motor control and provide cues for designing interventions aimed at alleviating the functional burdens of acquired injuries of the central nervous system.

## Graphical abstract

Human cortico‐cerebellar interactions during motor error correction were explored using synchronized cerebellar and EEG recordings. Results highlight centro‐parietal error positivity, fronto‐centro‐parietal desynchronization, and alpha/low‐beta cortico‐cerebellar coherence, underscoring bidirectional neural interactions in motor error processing.

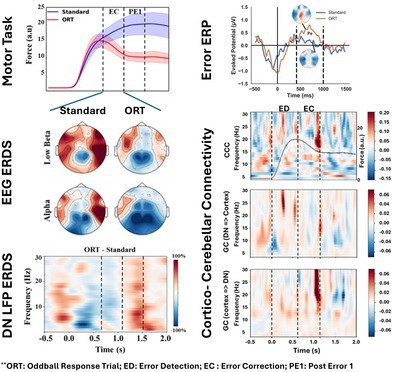

## 1 Introduction

Motor error processing—the brain's ability to detect and correct discrepancies between intended and actual movements—is essential for adaptive motor control and learning (Shadmehr and Krakauer 2008). The cerebellum plays a critical role in this process acting as a forward internal model to predict the outcomes of motor commands, compare those predictions with sensory feedback, and update the model to improve future predictions (Wolpert et al. 1995; Shadmehr and Mussa‐Ivaldi 1994). This predictive coding mechanism enables precise movement adjustments and error correction, which are vital for skilled motor performance (Manto et al. 2012). Error correction mechanisms have been previously investigated with electroencephalography (EEG) event‐related potentials (ERPs) and event‐related desynchronization/synchronization (ERD/S) (Gehring et al. 1993; Falkenstein et al. 2000; Pfurtscheller and da Lopes Silva 1999; Pfurtscheller et al. 2005). ERPs, such as the Error‐Related Negativity (ERN) and Error Positivity (Pe), provide insights into the cortical temporal dynamics in response to errors (Holroyd and Coles 2002; Hajcak et al. 2005), whereas ERD/S studies highlight oscillatory changes associated with error processing (Pfurtscheller et al. 2005). While the cerebellum's role in predictive coding and motor control has been documented (Wolpert et al. 1995; Manto et al. 2012), there is a significant gap in our understanding of how cortico‐cerebellar interactions contribute to error detection and correction. In particular, there is a paucity of data from human physiology of the deep cerebellar nuclei and their reciprocal interactions with the cerebral cortex during motor planning and execution. Addressing this gap could provide crucial mechanistic insights and guide therapeutic approaches for motor rehabilitation.

While cerebral cortical areas involved in motor error processing can be examined with noninvasive electrophysiological techniques, such as EEG and magnetoencephalography (MEG), assessment of cerebellar activity in humans is challenged by the limited signal to noise ratio and spatial resolution of EEG and MEG (Andersen et al. 2020). As a result, data characterizing human cerebellar and cortical connectivity during movement are limited. We present here the first in‐human examination of cerebellar dentate nucleus (DN) electrophysiology and cortico‐cerebellar connectivity during motor error processing, enabled using invasive recordings from the DN and EEG. The works were conducted in individuals enrolled in a phase I clinical trial investigating the effects of deep brain stimulation (DBS) of the cerebellar dentate nucleus for chronic post‐stroke motor rehabilitation. Participants performed a task comprising a mixture of standard and oddball trials, where oddball trials involved unanticipated changes in the amount of force generation required to complete the task and elicited secondary error correction efforts to achieve trial success. By analyzing ERP components, ERD/S in EEG, DN local field potentials (LFPs), and cortico‐cerebellar coherence, we aimed to elucidate how the human dentatothalamocortical pathway participates in error detection and correction in real time. The characterization of these electrophysiological findings can serve as foundational knowledge to develop targeted interventions, such as neurostimulation protocols (Fleury et al. 2021) or rehabilitation strategies (Leech et al. 2022; Kumar et al. 2019), aimed at improving motor recovery after acquired brain injuries and for disorders of the cerebellothalamocortical pathway.

## 2 Materials and Methods

### 2.1 Participants

**FIGURE 1:**
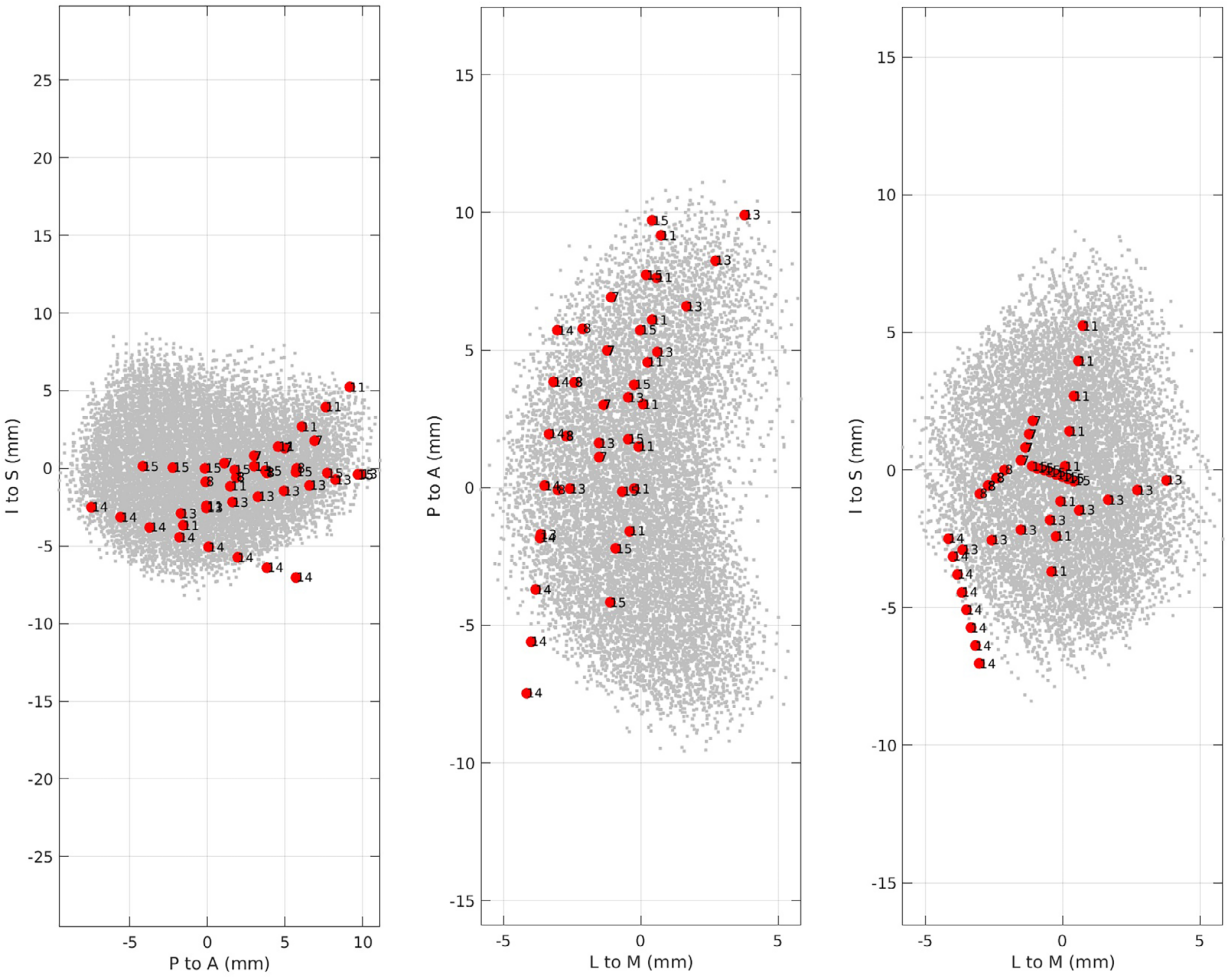
Implanted DN from each participant (shown in gray dots) aligned relative to their principal axes (inferior to superior, I to S; posterior to anterior, P to A; lateral to medial, L to M). Localization of DBS lead contacts (red colored dots) in the DN is labeled with the participant ID number.

Data were derived from participants undergoing a single‐site, phase I clinical trial (Clinicaltrials.gov NCT02835443) investigating the safety and feasibility of DN DBS in chronic, post‐stroke motor rehabilitation (Baker et al. 2023). The study was monitored under an Investigational Device Exemption from the US Food and Drug Administration, with all local research activities approved by the Cleveland Clinic Institutional Review Board and participants providing written informed consent. All recruitment and data collection activities happened between June 2016 and Oct 2020. All participants underwent surgery for implantation of a single DBS lead (Vercise, Boston Scientific, Valencia, California USA) in the DN contralateral to the stroke‐affected cerebral hemisphere. Out of the six participants reported in the current study, two were implanted with the Vercise Cartesia 8‐contact directional lead, which consists of a conical distal contact, a proximal annular contact, and two levels of three radially arranged directional contacts in‐between (contact length:1.5 mm, contact spacing: 2 mm, contact span: 7.5 mm). The remaining participants had a linear 8‐contact lead that consists entirely of annular contacts (contact length: 1.5 mm, contact spacing: 2 mm, contact span: 15.5 mm). Figure 1 shows the localization of the contacts in the DN for each participant in axial, coronal, and sagittal planes.

### 2.2 Data Acquisition

Data collection procedures are detailed elsewhere (Gopalakrishnan et al. 2022). Briefly, electrophysiological data were collected during a 5–7‐day externalization period immediately following surgical implantation of the DBS lead. LFP data were recorded from the DN by means of a temporary, percutaneous extension attached to the implanted DBS lead. A 36‐channel EEG system with conventional Ag/AgCl cup electrodes was positioned according to the standard 10–20 protocol to record scalp EEG data, with additional electrodes placed at: “AF7”, “AF3”, “AFz”, “AF4”, “AF8”, “FT9”, “FC5”, “FC1”, “FC2”, “FC6”, “FT10”, “TP9”, “CP5”, “CP1”, “CP2”, “CP6” & “TP10”. All LFP and EEG data was online referenced to Pz. The skin to electrode impedance was maintained below 5 kΩ. BrainAmp DC amplifier with BrainVision Recorder software (Brain Products) was used to collect the electrophysiological data at a sampling rate of 5000 samples per second with a high pass filter set at 0.1 Hz and built‐in low pass filter at 1KHz. Grip force was measured using a strain gauge‐based isometric hand dynamometer (model HDBTA, Vernier) connected to a Powerlab interface (model PL3516‐0158, ADinstruments) at a sampling rate of 1000 samples per second. Data were stored for subsequent analysis using LabChart (v8.0, AD Instruments).

### 2.3 Task and Behavior

Participants squeezed a handheld dynamometer with their paretic hand to move a 2D ball displayed on a computer screen in response to a visual “Go” cue. The task required them to exert either 40% of their maximum voluntary contraction (MVC) for standard trials or 20% MVC for oddball trials, which were randomly presented at a ratio of 7:3. The 40% and 20% MVC thresholds were chosen to balance task difficulty and minimize fatigue, ensuring a sufficient effort difference for motor‐related EEG and LFP analysis. The 7:3 ratio of oddball to standard trials was selected to maintain unpredictability and attentional challenge, while ensuring enough trials for robust analysis, consistent with Go/No Go task designs (Littman and Takács 2017).

They were instructed to respond to the “Go” cue and reach the stationary target with the 2D ball as fast as possible by applying force to the dynamometer and holding the grip for 2 s. On average, participants completed 239 ± 49 randomized trials.

**FIGURE 2:**
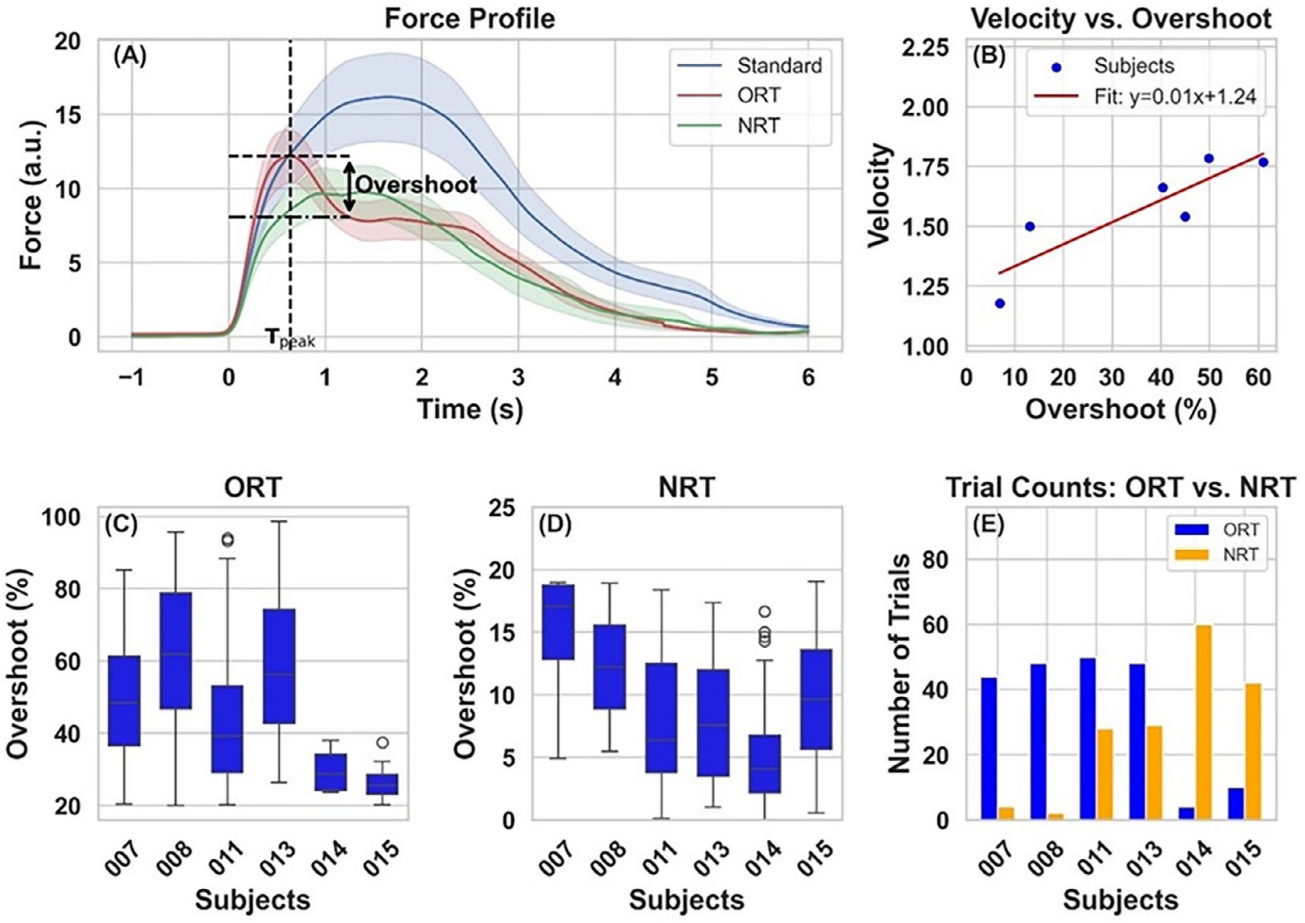
Force control and response dynamics. (A) Trajectory and force profile: 2‐D ball trajectories and force profiles for standard, oddball response trials (ORT), and oddball no response trials (NRT), showing greater overshoot in ORT. Dotted line (*T*_peak_) marks the time point when peak force is reached in ORT. (B) Velocity vs. Overshoot: Relationship between overshoot percentage and response velocity (force per unit time), indicating that higher velocity is linked to increased overshoot. (C) Overshoot distribution for ORT: Distribution of overshoot percentages (> 20%) for ORT across participants. Box plot shows the median and interquartile range and error bars show the min and max values. (D) Overshoot distribution for NRT: Distribution of overshoot percentages (< 20%) for NRT across participants. (E) Response classification: Bar plot depicting the number of ORT and NRT for each of the six participants.

During oddball trials, participants tended to overshoot the target before readjusting their grip to successfully meet the specified target. The overshoot was quantified as a percentage ratio of the overshoot value relative to the 20% MVC oddball target. Oddball trials were categorized based on the motor behavior exhibited during the task, using overshoot as the criterion as per prior studies (Shalgi et al. 2009; Eimer 1996; Balzus et al. 2023). Trials where the overshoot exceeded 20% were termed oddball response trials (ORT), while trials where the overshoot was less than 20% were termed no response trials (NRT). Figure 2A shows the mean ± SEM for each condition, highlighting overshoot in ORT, with *T*_peak_ marking the peak force time point. Figure 2B illustrates the relationship between the overshoot percentage and the velocity of the 2‐D ball. A positive correlation (*p* < 0.05) indicates that a higher velocity, associated with a faster grip on the dynamometer, was more likely to result in an overshoot during oddball trials. Figure 2C,D show the distribution of ORT and NRT trials, while Figure 2E displays the number of trials classified as ORT and NRT for all participants.

On average, participants had 34 ± 21 ORT, 28 ± 22 NRT, and 178 ± 36 standard trials. Previous research (Olvet and Hajcak 2009; Wang et al. 2015) has suggested that a minimum of 10 ORT and NRT are required to consider a participant as responsive or nonresponsive to oddball trials, respectively. Consequently, we included only participants who had at least 10 trials for each response category. Following this approach, ORT were analyzed for 4 subjects (007, 008, 011, and 013) while NRT were analyzed for 4 subjects (011, 013, 014, and 015). To compare ORT and NRT with standard trials, we created two random subsets of standard trials that matched the number of ORT and NRT for each participant.

### 2.4 EEG and LFP Preprocessing

EEG and LFP data preprocessing was conducted using the MATLAB‐based FieldTrip toolbox (Oostenveld et al. 2011) and custom scripts. EEG data were manually cleaned of artifacts from compensatory movements, segmented into trials (−3 to 6 s relative to movement onset), and filtered to remove line noise using discrete Fourier transform or spectral interpolation. The data were then downsampled to 200 samples per second, cleaned of muscle, EKG, and ocular artifacts via independent component analysis, and converted to scalp current densities using a surface Laplacian approach. EEG data from left hemisphere stroke patients were flipped left–right to align with a common (right) side corresponding to the affected hemisphere. For LFP data, the same segmentation (−3 to 6 s relative to movement onset) and noise removal procedures were applied. The ipsilesional cortex was defined by eight electrodes: “F4”, “F8”, “FC2”, “FC6”, “C4”, “CP2”, “CP6”, and “P4”, while the central region was defined by three electrodes: “Pz”, “Fz”, and “Cz”. Additionally, the fronto‐central region included “FC2”, “FC1”, “Cz”, and “Fz”, while the centro‐parietal region consisted of “CP2”, “CP1”, “Cz”, and “Pz”. The DN LFPs were analyzed as bipolar configurations derived from DBS lead contacts: participants with an eight contact linear lead had seven bipolar channels (LFP1‐2, LFP2‐3, LFP3‐4, LFP4‐5, LFP56, LFP6‐7, LFP7‐8), while those with eight‐contact directional leads had nine bipolar channels (LFP1‐2, LFP1‐3, LFP1‐4, LFP2‐5, LFP3‐6, LFP4‐7, LFP5‐8, LFP6‐8, LFP7‐8).

### 2.5 ERP Analysis

ERP data analysis was performed using MNE‐Python version 1.7.1 (Gramfort et al. 2013). The data trials were bandpass filtered from 0.1 to 30 Hz using a finite impulse response (FIR) filter. The preprocessed data trials were re‐epoched to include 1000 ms before and 2000 ms after movement onset. Trials were baseline corrected and averaged separately for standard, ORT, and NRT conditions. Error processing research has shown that the Pe component is a positive deflection ERP with a centro‐parietal distribution, typically occurring 200–800 ms after an incorrect response (Shalgi et al. 2009). Therefore, ERPs for the centro‐parietal region were computed by averaging recordings from CP2, CP1, Cz, and Pz electrodes. The Pe component was identified as a slow, positive peak occurring between 200 and 800 ms over the centro‐parietal region. A nonparametric permutation test was performed to assess whether the Pe component was significantly greater in the ORT than NRT category in comparison to standard trials.

### 2.6 ERD/S Analysis

The preprocessed EEG and LFP data, after artifact removal, were converted into time‐frequency representations using Morlet wavelets with the number of cycles in the wavelet as one per frequency, covering a frequency range from 1 to 30 Hz in 1 Hz increments. Event‐related power changes were assessed by averaging the normalized power across all time points within each frequency bin, relative to the average power of that frequency throughout the experimental session. Power decreases (below zero) were identified as event‐related desynchronization (ERD), while power increases (above zero) were identified as event‐related synchronization (ERS). In humans, sensorimotor ERD/S in the alpha (mu rhythm, 8–13 Hz) and beta bands (15–30 Hz) reflects cortical activity associated with motor functions (Toro et al. 1994). To analyze ERD/S in motor error processing, we divided the frequency bands into four distinct bands: theta (4–7 Hz), alpha (8–12), low beta (13‐20 Hz) and high beta (21–30 Hz).

To analyze time‐related changes in ERD/S, we divided the time course into four distinct phases: 1. Error Detection (ED): This phase spans from 0 s to *T*_peak_, where *T*_peak_ represents the moment when the peak force value is reached in ORT (see, Figure 2A). 2. Error Correction (EC): This interval extends from *T*_peak_ to 1.1 s, with 1.1 s marking the average time when the force profile in ORT meets the actual target (i.e., 20% MVC). The EC phase is crucial as it shows how participants adjusted their motor output to correct the initial overshoot. 3. Post Error 1 (PE1): This phase covers the period from 1.1 s to 1.5 s following the error correction. 4. Post Error 2 (PE2): This final phase ranges from 1.5 s to 2 s after the error. By dividing the time into four phases, we were able to assess the temporal dynamics of ERD/S changes related to error detection and correction.

### 2.7 CCC/GC Analysis

We computed the CCC for each combination of the DN LFP channel and scalp EEG electrode over central and ipsilesional regions, after partializing for the contribution of the EMG. Coherence in the frequency domain quantifies the consistency of the phase difference and amplitude across trials between two signals. CCC was computed across time (−500–2000 ms in steps of 5 ms) and frequency (1 to 30 Hz, in steps of 1 Hz) with coefficients derived using Morlet wavelets. A coherence value of 0 indicates that the signals being compared have no consistent linear phase relationship, whereas a value of 1 indicates that the two signals are fully phase coherent.

We then computed the difference in CCC by subtracting the CCC of standard trials from the oddball trials (ORT and NRT). For each participant, we employed a nonparametric cluster‐based permutation approach (Gopalakrishnan et al. 2022) to analyze the CCC difference maps to identify the DN LFP channel that exhibited the largest coherence (*p* < 0.05, FDR corrected) over the central and ipsilesional EEG channels across four frequency bands: high beta (21–30 Hz), low beta (13–20 Hz), alpha (8–12 Hz), and theta (4–7 Hz). The analysis focused on the period between 0 and 1.5 s, covering the ED, EC, and PE1 phases. This approach revealed one DN LFP channel and two neighboring EEG channels over the central and ipsilesional cortex that exhibited the largest coherence cluster. To further investigate directional interactions, we performed a nonparametric spectral GC analysis (Dhamala et al. 2008) between the DN and the central and ipsilesional cortices, utilizing the channels identified in the above analysis.

### 2.8 Statistical Analysis

To investigate differences between standard and oddball trials in ERD/S, CCC, and GC, generalized linear mixed effects models (GLMM) were used (Fromer et al. 2018). GLMM are flexible extensions of linear regression, which can account for non‐Gaussian response distributions, as well as repeated measurements per subject and hierarchical/nested data (e.g., trial nested within contact within subject), without having to average item‐level data to a single response per subject. Models were built separately for each frequency band and each time course phase. Each model included random effects for contact nested within subject and a fixed effect for trial type (standard versus oddball). A compound symmetry covariance matrix was implemented. Mahalanobis distance by chi‐square plots were used to evaluate multivariate normality. Both ERD/S and CCC were multivariate normal, so the linear model was used and each model parameter (*β*) with 95% confidence intervals represents the adjusted mean difference between standard and oddball trials, while accounting for all sources of clustering in the data. GC data were multivariate lognormal; however, these models were underpowered and thus all GC data were interpreted qualitatively and without drawing statistical conclusions. All models were fit using SAS Studio v.3.81 with significance indicated by *p* < 0.05 for the model parameter for trial type. Fit was evaluated using Bayes Information Criterion and visual examination of residuals.

## 3 Results

### 3.1 Participant Characteristics

Data presented in this study were acquired from 6 participants in a phase‐I clinical trial that tested the efficacy of DN DBS for post‐stroke rehabilitation. Participants suffered from moderate‐to‐severe upper extremity hemiparesis after middle cerebral artery ischemic stroke but had sufficient residual motor function to engage in the motor tasks required in the study. The participant demographics are summarized in Table 1. The mean age was 57.3 ± 7.21 years (48–70 years of age). One participant was female, and three had strokes on the right hemisphere affecting their left extremity.

**TABLE 1:** Participant characteristics.

Participant	Age (years)	Sex	Affected extremity	Dominant extremity	Years after stroke	Impairment level			
						UEFM Total (Max 66)	UEFM Hand (Max 30)	Avg. AMAT‐FA	Avg. AMAT‐QOM
007	57	M	R	R	1.8	18	5	1.6	1.6
008	70	F	R	R	1.9	26	10	3.8	3.7
011	48	M	R	R	1.7	38	12	2.8	2.9
013	54	M	L	R	2	23	7	2.9	3.0
014	54	M	L	L	3.4	25	7	2.7	2.8
015	69	M	L	L	3.2	24	9	3.1	3.0

### 3.2 Error Positivity Component in ORT

**FIGURE 3:**
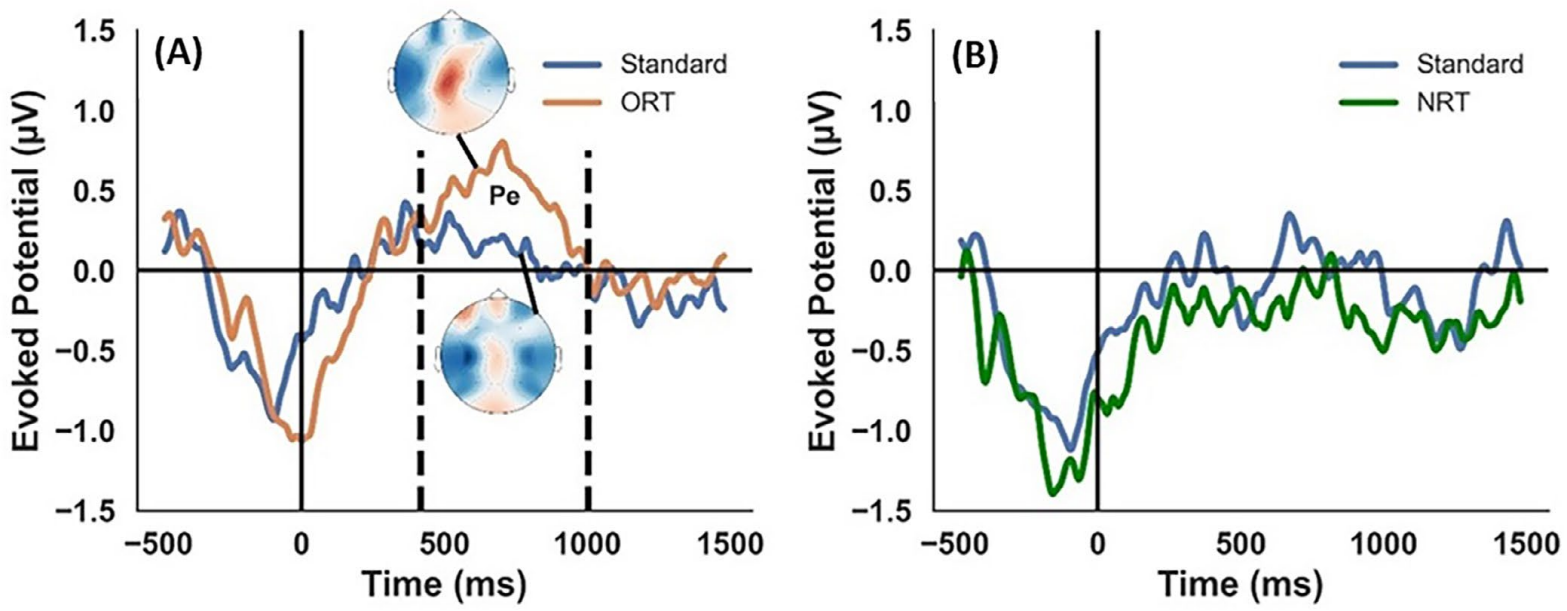
Centro‐Parietal ERP Differences in ORT Versus Standard Trials. (A) centro‐parietal ERPs for ORT vs standard trials, time‐locked to stimulus onset and grand averaged across all participants. The Pe component, highlighted between 417 and 1002 ms (dashed vertical lines), demonstrates a significant difference between ORT and standard trials (Cluster based permutation test, *p* < 0.05). The time‐averaged topoplot (top) shows higher ERP amplitudes for ORT compared to standard trials (bottom) in the centro‐parietal region. (B) ERPs for NRT versus standard trials, time‐locked and grand averaged, show no significant differences.

A nonparametric cluster‐based permutation test comparing the ERPs derived from the centro‐parietal region between standard and ORT revealed significant ERP differences between oddball and standard trials in the epoch defined between time 417 and 1002 ms (Figure 3A). The test indicated that the ERP for ORT (0.398 ± 0.089 μV) was significantly higher (*p* = 0.0049) than that of standard trials (0.006 ± 0.081 μV), suggesting the presence of an error positivity (Pe) component. In contrast, there was no difference between the NRT (control) and standard trials (Figure 3B).

### 3.3 ERD/S Results From EEG

**FIGURE 4:**
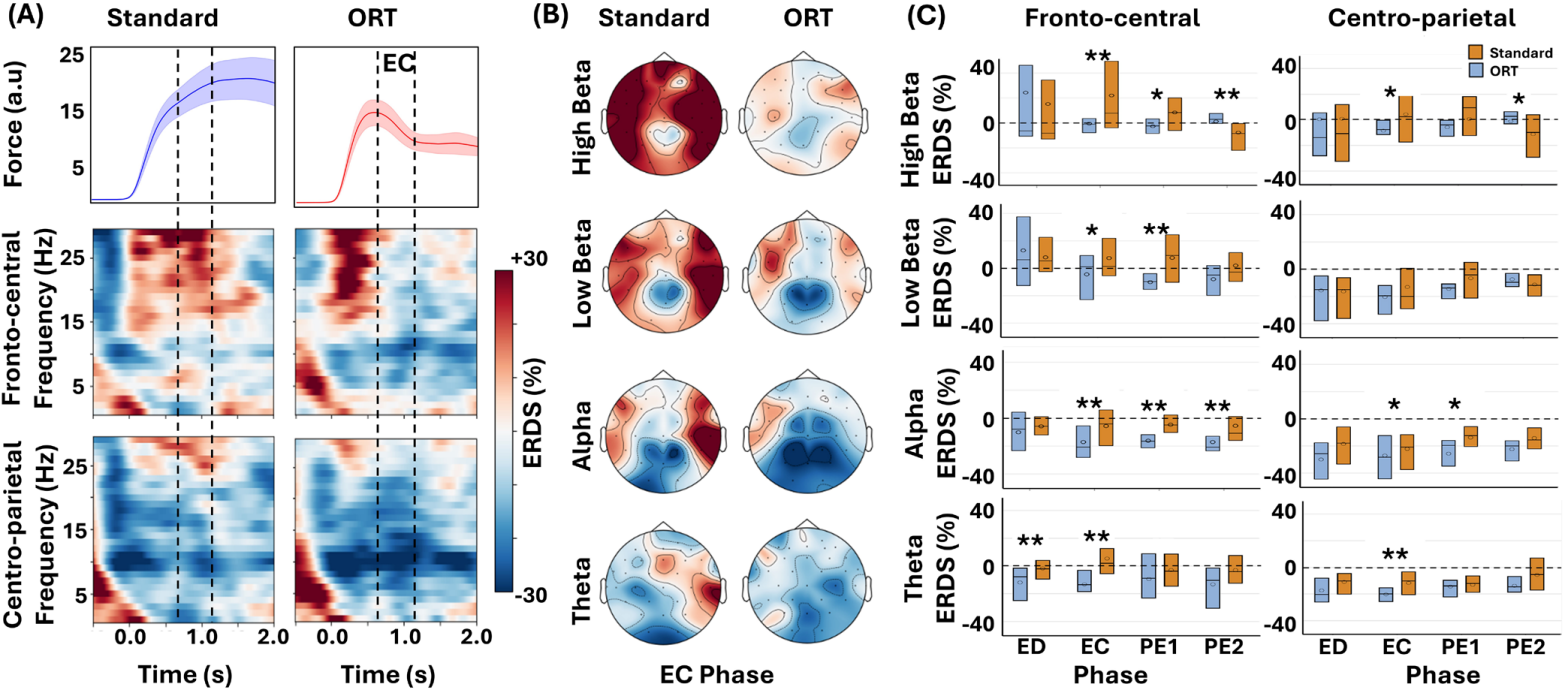
Comparison of EEG ERD/S between standard and ORT. (A) Top: Force profiles (mean and SEM) for standard and ORT, time duration between lines highlights the error correction (EC) phase during ORT. Middle: Averaged time‐frequency plots from fronto‐central EEG channels, showing ERD/S dynamics for both standard and ORT. Bottom: Averaged time‐frequency plots from centro‐parietal channels, illustrating ERD/S patterns across the two conditions. Dotted lines highlight the error correction (EC) phase. (B) Topoplot representing ERD/S averaged across the EC phase (670–1100 ms) for standard and ORT, showing spatial distribution differences between conditions. (C) Time‐stratified comparison of ERD/S across four distinct phases: ED, EC, PE1, and PE2 analyzed in theta, alpha, low beta, and high beta bands for both standard and ORT, highlighting statistical differences. **p < 0.01, *p < 0.05.

Grand averaged ERD/S plots across all participants in the time‐frequency domain for the fronto‐central and centro‐parietal regions revealed greater ERD during the EC phase in the ORT (fronto‐central: −9.10% ± 17.98%, centro‐parietal: −18.42% ± 17.25%) compared to the standard (fronto‐central: 2.00% ± 28.09%, centro‐parietal: −14.38% ± 23.39%), across all four frequency bands (Figure 4A). Frequency band‐specific topoplots of ERD/S averaged across the EC further highlighted that motor error‐related ERD was predominantly associated with the central region across all four frequency bands (Figure 4B).

In the fronto‐central region, the generalized linear mixed effects models (GLMM) analysis revealed significantly greater ERD during EC across all four frequency bands—theta (*β* = −0.1867; 95% CI −0.3035, −0.0699; *p* = 0.003), alpha (*β* = −0.1146; 95% CI −0.1870, −0.0421; *p* = 0.003), low beta (*β* = −0.1185; 95% CI −0.2070, −0.0299; *p* = 0.011), and high beta (*β* = −0.2268; 95% CI −0.3553, −0.0982; *p* = 0.001) during ORT as compared to standard trials (Figure 4C, column 1). In the PE1 phase, significantly greater ERD was observed only in the alpha (*β* = −0.1147; 95% CI −0.1752, −0.0542; *p* = 0.0006) and beta bands (low beta *β* = −0.1776; 95% CI −0.2735, −0.0816; *p* = 0.0008; high beta *β* = −0.1105; 95% CI −0.2135, −0.0075; *p* = 0.036). In the PE2 phase, significantly greater ERD was observed only in the alpha band (*β* = −0.1165; 95% CI −0.1900, −0.0430; *p* = 0.003). No difference was observed in the ED phase.

In the centro‐parietal region, the GLMM analysis revealed significantly greater ERD during the EC phase across theta (*β* = −0.0868; 95% CI −0.1504, −0.0232; *p* = 0.009), alpha (*β* = −0.1148; 95% CI −0.2098, −0.0198; *p* = 0.02), and high beta (*β* = −0.1278; 95% CI −0.2413, 0.0142; *p* = 0.029) frequency bands (Figure 4C, column 2) in ORT versus standard trials. In the PE1 phase, significantly greater ERD was observed in the alpha band (*β* = −0.1181; 95% CI −0.2099, −0.0262; *p* = 0.014). When comparing the ERD/S of EEG in the fronto‐central and centro‐parietal regions between the NRT and standard conditions, we found no significant differences across any phase or frequency band.

### 3.4 ERD/S Results From DN

**FIGURE 5:**
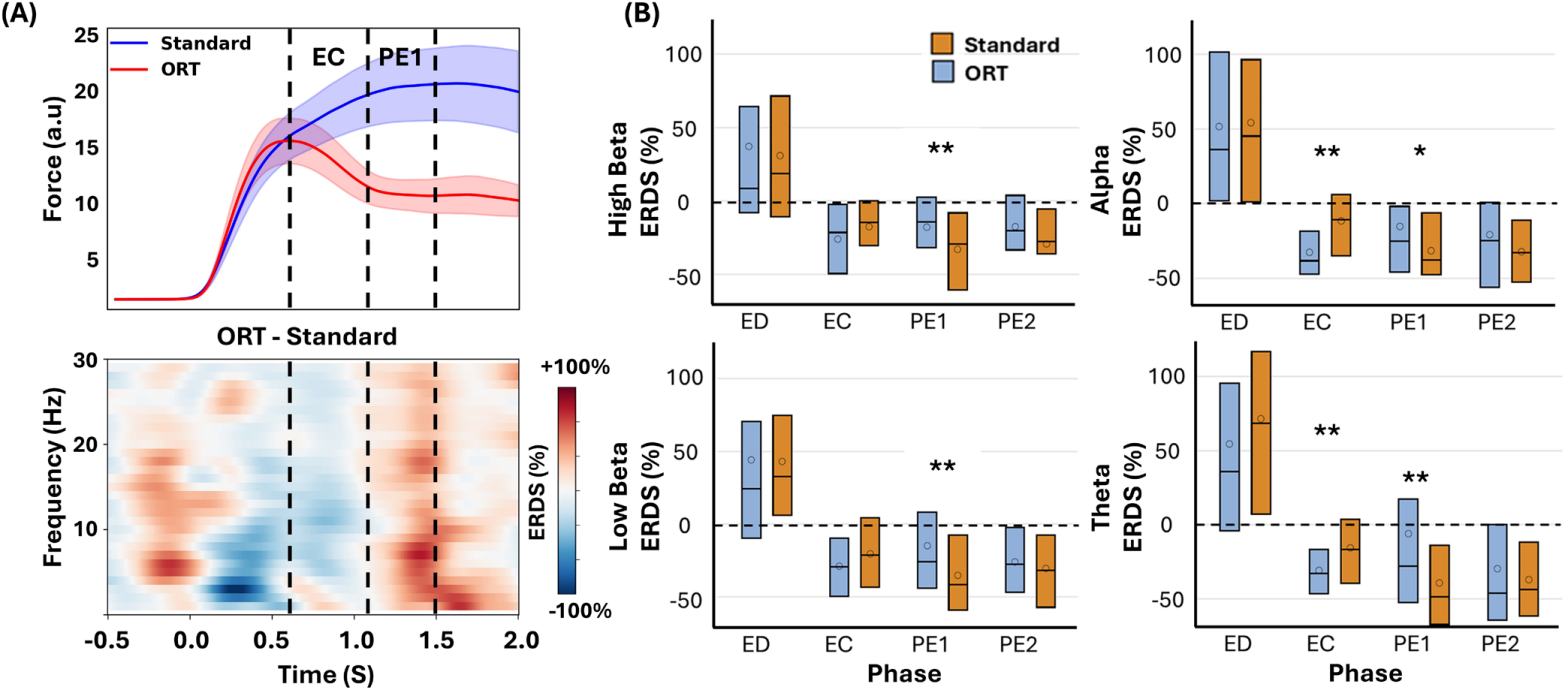
Comparison of DN ERD/S between standard and ORT. (A) Top: Force profiles (mean and SEM) for standard and ORT. Dotted lines highlight the EC and PE1 phases. Bottom: Averaged time‐frequency difference plot (ORT—standard) from DN contacts, showing ERD/S dynamics. (B) Time‐stratified comparison of ERD/S across four distinct phases: ED, EC, PE1, and PE2 in theta, alpha, low beta, and high beta bands for both standard and ORT, highlighting statistical differences. **p < 0.01, *p < 0.05. Values to derive box plots are adjusted to account for subject‐level random effects.

Consistent with the EEG, LFPs from all DN channels also demonstrated greater desynchronization during the EC phase in ORT (−22.91% ± 34.89%) compared to standard trials (6.78% ± 22.00%) (Figure 5A). In contrast, during the post error 1 (PE1), the DN LFPs showed less desynchronization in ORT (−14.71% ± 52.55%) compared to the standard (−43.57% ± 28.37%). GLMM analysis revealed significantly greater ERD in the ORT compared to the standard in the EC phase in the theta (*β* = −0.1508; 95% CI −0.2456, −0.0561; *p* = 0.002) and alpha bands (*β* = −0.2083; 95% CI −0.2957, −0.1208; *p* < 0.0001). In the PE1 phase, significantly less ERD was observed in the ORT compared to the standard across all frequency bands (theta *β* = 0.3323; 95% CI 0.1346, 0.5300; *p* = 0.001; alpha *β* = 0.1647; 95% CI 0.0235, 0.3059; *p* = 0.023; low beta *β* = 0.2002; 95% CI 0.0597, 0.3407; *p* = 0.006; high beta 0.1494; 95% CI 0.0372, 0.2615; *p* = 0.01) (Figure 5B). When comparing the ERD/S of DN LFP between the NRT and standard, no significant differences were observed across any time phase or frequency band.

### 3.5 Increased Cortico‐Cerebellar Coherence (CCC) and Granger Causality (GC) During Error Correction

**FIGURE 6:**
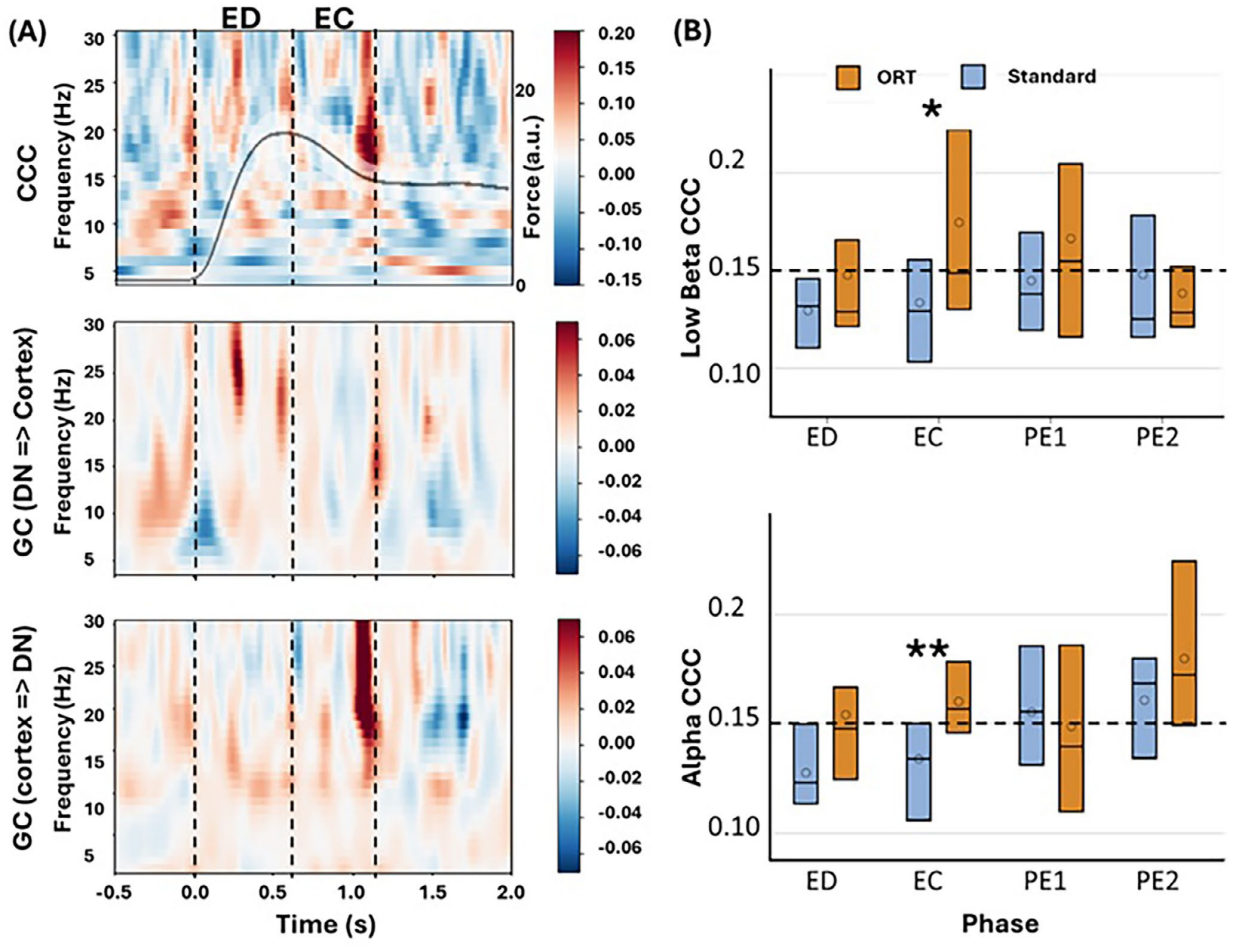
CCC/GC comparison between standard and ORT. (A) Top: Grand averaged CCC difference plot (ORT—standard) with ORT force profile (black line, right axis), illustrating higher CCC during the ED and EC phases for ORT compared to standard. Middle: Grand averaged GC difference plot (ORT—standard) directed from DN to ipsilesional cortex, illustrating higher causal influence of DN over ipsilesional cortex during both ED and EC. Bottom: Grand averaged GC difference plot (ORT—standard) directed from ipsilesional cortex to DN, illustrating higher causal influence of ipsilesional cortex over DN during EC only. (B) Time‐stratified comparison of CCCs across four distinct phases: ED, EC, PE1, and PE2 in alpha and low beta bands illustrating significant differences between standard and ORT. No significant differences were observed in the theta and high beta bands. **p < 0.01 and *p < 0.05.

For the CCC/GC analysis, we first used a data‐driven approach to identify the LFP‐EEG pairs that exhibited the largest coherence in each participant separately for ORT and NRT trials, as detailed in Methods Section 2.7. No significant clusters were found in the NRT trials. Grand averaged CCC difference plots (ORT—standard) across all participants in the time‐frequency domain revealed greater coherence during EC in the ORT (0.16 ± 0.04) compared to the standard trials (0.13 ± 0.03) (Figure 6A top). Specifically, during the EC phase, CCC in the alpha (*β* = 0.0232; 95% CI 0.0084, 0.0441; *p* = 0.008) and low beta bands (*β* = 0.041; 95% CI 0.0012, 0.0808; *p* = 0.045) was significantly higher in the ORT compared to the standard (Figure 6B). Further analysis of GC revealed that increased CCC during EC in the ORT was marked by bidirectional interaction between DN and the ipsilesional cortex (Figure 6A, center and bottom panel). We also observed increased CCC in the low and high beta bands during the error detection (ED) in the ORT, which was marked by only DN to ipsilesional directed increase in GC; however, there was no statistical significance.

## 4 Discussion

We used invasive LFP recordings derived from DBS leads implanted in the cerebellar DN to investigate its role in motor error processing during a standard oddball task. Oddball trials were further split as ORT and NRT respectively based on whether the participants were responsive or not responsive to the covert perturbation. We analyzed EEG ERPs, ERD/S in EEG and DN LFPs, and CCC/GC to understand how the DN contributes to error monitoring and correction. We found increased Pe and greater desynchronization and coherence in specific frequency bands during the ORT but not in NRT, indicating enhanced cortico‐cerebellar activity during error processing. To our knowledge, this is the first study to report DN electrophysiology and CCC during motor error processing with data directly recorded from DN in human participants.

### 4.1 The Role of Error Positivity Observed in ORT

The significantly larger Pe observed in the centro‐parietal region during the ORT is consistent with prior research linking the Pe to conscious error detection and internal awareness of errors (Davies et al. 2001; Butterfield and Mangels 2003). The presence of the Pe without noticeable ERN in the ORT highlights the Pe's role in processing discrepancies between intended and actual responses, even in the absence of explicit feedback or knowledge of the correct response. This observation supports the notion that the Pe can occur independently of the ERN, reflecting a distinct mechanism for conscious error processing (Di Gregorio et al. 2018; Shalgi et al. 2009). Specifically, while the ERN is often associated with early automatic error detection, the Pe represents a later, more conscious recognition of errors, which aligns with findings from our study where participants adjusted their responses to unexpected changes without clear external cues. This distinction supports the Pe's role in conscious error awareness, separate from the automatic processes reflected by the ERN.

### 4.2 ERDS During Error Processing

Our findings reveal increased ERD during the EC phase across theta, alpha, and beta bands, with more pronounced ERD in the alpha and beta bands during post‐error phases in central regions. Significant alpha and beta ERD in these phases highlight heightened cortical excitability, which is crucial for motor control and adaptation (Pfurtscheller and da Lopes Silva 1999; Takemi et al. 2013). Notably, the scalp‐level results are largely consistent with prior studies on healthy controls (Takemi et al. 2013; Tan et al. 2014), suggesting that despite stroke, the general patterns observed in the scalp recordings align with findings from healthy subjects. The increased ERD observed in the central region during the EC phase of the ORT could reflect activity in the anterior cingulate cortex (ACC) which is crucial for monitoring errors and adjusting behavior (Holroyd and Coles 2002; Carter et al. 1998). Previous research from our group (Gopalakrishnan et al. 2022) demonstrated that movement‐related oscillations in the DN mirrored those in the sensorimotor cortex, with ERD in one area correlating with ERD in the other across alpha and low beta bands during phasic movement. In this study, focusing on error processing, we found similar patterns in alpha, beta, with prominent ERD also in the theta band, in both EEG and DN LFP ERD. Our findings further iterate the strong coupling between the cerebral cortex and DN in the alpha and low beta band during motor error processing.

### 4.3 Cortico‐Cerebellar Connectivity

Our results reveal enhanced connectivity between the DN and ipsilesional cortex during ED and EC in the ORT compared to standard trials. Specifically, CCC was significantly higher during EC in the ORT in the alpha and low beta bands. This suggests stronger synchrony between the DN and ipsilesional cortical regions during error processing. These findings align with our previous study on dynamic motor control during movement onset and offset (Gopalakrishnan et al. 2022) and with other research on normal states in nonhuman primates (Soteropoulos and Baker 2006). Increased GC from the DN to the ipsilesional cortex during the ED phase could be indicative of the cerebellum sending motor commands to the ipsilesional cortex. Upon receiving commands from DN, during EC, increased GC from the ipsilesional cortex to DN could be indicative of the feedback from the cortex to DN. These interactions align with the well‐established model that the cerebellum initially predicts the outcomes of motor commands, then compares these predictions with sensory feedback and receives inputs from the cerebral cortex to update its internal model to refine future predictions.

### 4.4 Implications for Stroke Rehabilitation

Error‐related neural responses, such as the Pe and oscillations in alpha and beta bands, play a critical role in detecting and correcting errors, which is vital for motor adaptation and learning (Yordanova et al. 2024). The enhanced cortico‐cerebellar connectivity and the observed desynchronization of oscillatory activity during error processing in our study suggest that these neural responses can be targeted to improve rehabilitation outcomes. With the emergence of closed‐loop systems (Gilron et al. 2021), there is a growing need for reliable electrophysiological feedback signals that can trigger stimulation delivery. Unlike Parkinson's disease or dystonia where LFP signals from subthalamic nucleus (STN) and globus pallidus pars interna (GPi) respectively encode disease severity (Asadi et al. 2025), biomarkers correlating with stroke symptoms and severity are still lacking. Our findings provide preliminary evidence for the role of alpha and low beta oscillations in the cerebellum that could serve as valuable feedback for guiding DN DBS interventions. Further, incorporating real‐time neurophysiological feedback, such as the Pe and cerebellar‐cortical coherence, into robotic‐assisted therapies offers a dynamic approach, allowing for individualized adjustments based on the patient's performance (Kumar et al. 2019). Moreover, strengthening the feedback loops between the cerebellum and the motor cortex could lead to more effective motor control and adaptation during rehabilitation exercises (Daskalakis et al. 2004; Seidler et al. 2013). These findings support the integration of error‐based learning principles into stroke rehabilitation, potentially leading to better recovery and functional outcomes.

## 5 Limitations

We acknowledge the low sample size of our dataset; however, the size of the sample is compensated by the rich and novel data set, consisting of the first‐ever direct electrophysiological examination of the human DN, combined with EEG, during a motor error processing task. Our participants had strokes in different locations and of varying intensities which might have influenced the results. However, the six participants studied here are homogeneous in terms of impairment level and functionality. Further, the data presented here were collected within 1 week of the surgical implantation of the DN DBS leads which might have had adverse effects on the attention and alertness of the participants. This could explain why our participants had so many NRT. However, we turned this to our advantage by comparing the NRT with ORT clearly illustrating the non‐participation of the cerebellum when the error was simply omitted.

## 6 Conclusions

In conclusion, our study provides novel evidence in support of the well‐established role of the cerebellum in predicting motor outcomes and refining prediction accuracy through feedback from the cortex. Our results further corroborate our earlier findings that DN and contralateral cerebral cortices work in tandem to achieve movement‐related goals. We show that this is not only true for motor control, but also for error processing. These results enhance our mechanistic understanding of the cerebellar role in error processing, which could lead to targeted neuromodulation therapies aimed at improving motor performance.

## Conflicts of Interest

Andre G. Machado and Kenneth B. Baker are consultants and have intellectual property licensed to Enspire DBS. Andre G. Machado is the chief medical/scientific officer of Enspire DBS. Enspire DBS funded part of the DN‐DBS clinical trial for stroke rehabilitation, but not the present research. This research was funded by the NIH BRAIN initiative.

## Data Availability

The data that support the findings of this study are available from the corresponding author upon reasonable request.
